# Bortezomib Use for a Critically Ill Patient with Angioimmunoblastic T-Cell Lymphoma

**DOI:** 10.1155/2022/6079633

**Published:** 2022-08-31

**Authors:** Motoharu Shibusawa

**Affiliations:** IMS Group, Shinmatsudo Central General Hospital, Department of Hematology, Chiba, Japan

## Abstract

Angioimmunoblastic T-cell lymphoma (AITL) accounts for 18.5% of all peripheral T-cell lymphomas. There is still no gold standard chemotherapy for treating newly diagnosed AITL. This case describes the use of bortezomib in newly diagnosed AITL. A 53-year-old man with no previous illness presented with erythema and swelling in the left neck. A diagnosis of AITL was made based on the results of lymph node biopsies. AITL progression led the patient to a severely deteriorated general condition. Bortezomib was thus administered, which resulted in a reduction in lymphadenopathies, the disappearance of tumor fever, and a decrease in serum lactate dehydrogenase levels. Subsequently, the patient's general condition gradually improved. Despite the patient's poor condition, bortezomib was well tolerated. After bortezomib administration, the patient did not require chemotherapy for approximately 10 months. The present case indicates that bortezomib is a possible treatment option for patients with AITL.

## 1. Background

Angioimmunoblastic T-cell lymphoma (AITL) accounts for 18.5% of all peripheral T-cell lymphomas, and it occurs frequently in Europe, Asia, and North America [1]. AITL is a neoplasm comprising mature *T* follicular helper cells. *T* follicular helper cells release various factors, including C-X-C motif chemokine ligand 13, interleukin (IL)-21, IL-10, IL-6, transforming growth factor-*β*, vascular endothelial growth factor (VEGF), and angiopoietin. A complex network of interactions exists between tumor cells and various cellular components in the reactive microenvironment [2]. The clinical presentations of AITL include lymphadenopathies, bone marrow involvement, skin rash, and laboratory test abnormalities (anemia, positive result on direct antiglobulin test, thrombocytopenia, hypergammaglobulinemia, and hypereosinophilia) [3]. Patients with AITL may also present with plasmacytosis [4–6].

To date, there is no gold standard chemotherapy for treating newly diagnosed AITL. AITL is usually treated with the CHOP regimen, which includes cyclophosphamide, doxorubicin, vincristine, and prednisone; CHOEP, which includes etoposide in addition to CHOP; and ACVBP, which includes doxorubicin, cyclophosphamide, vindesine, bleomycin, and prednisone. [3].

Bortezomib is an anticancer drug that inhibits the proteasome, inducing the accumulation of unfolded and misfolded proteins, which in turn leads to apoptosis and cell death. In addition, bortezomib inhibits the production of cytokines such as IL-6, insulin-like growth factor-1, and VEGF in bone marrow stromal cells. [7] Bortezomib is also an effective agent for the treatment of B-cell neoplasms, such as multiple myeloma, mantle cell lymphoma, and lymphoplasmacytic lymphoma. [7,8] Moreover, several previous studies have reported the efficacy of bortezomib for the treatment of T-cell neoplasms. [9–11].

This case report describes the successful use of bortezomib in a critically ill patient with AITL.

## 2. Case Presentation

**Figure 1:**
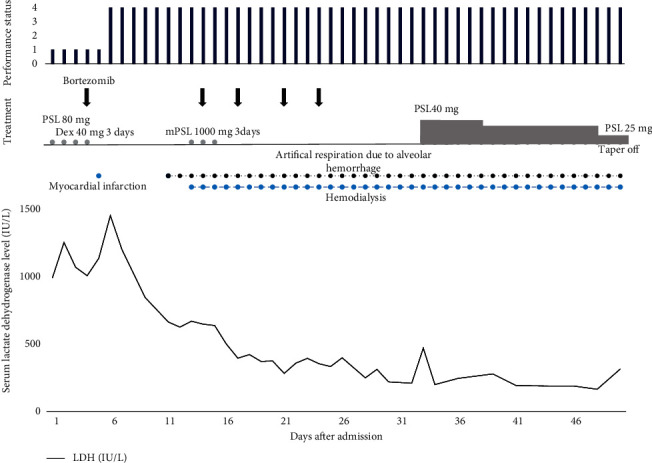
Clinical course after admission to our hospital. After bortezomib administration, the serum lactate dehydrogenase level decreased. LDH; lactate dehydrogenase. PSL; prednisolone, Dex:dexamethasone, and mPSL; methylprednisolone.

A 53-year-old man with no previous illness presented with erythema throughout the body 4 weeks before and with swelling in the left neck 6 weeks before admission to our hospital. The patient was admitted to another hospital 18 days before admission to our hospital. A whole-body computed tomography (CT) revealed lymphadenopathies and a ground-glass appearance in the lung. His soluble IL-2 receptor level was 5,445 U/mL. Prednisolone (PSL) administration was started at a dose of 30 mg daily. A left axilla lymph node biopsy was performed on the fourth day after another hospital admission. Then, the patient was transferred to our hospital. The clinical course after admission to our hospital is summarized in Figure 1. On the first hospital day, his consciousness was clear. His vital signs were as follows: body temperature, 38.3°C; blood pressure, 162/97 mmHg; pulse rate, 122 beats/min; and oxygen saturation, 91% in ambient air. Moreover, his Eastern Cooperative Oncology Group (ECOG) performance status was 1, and his Karnofsky scale score [12] was 80 points. Erythema in the face, arms, and trunk and palpable lymphadenopathies in the bilateral cervical and left axilla areas were noted. Regarding B symptoms, the patient had a fever of >38°C but no drenching night sweats or unintentional weight loss. Laboratory tests on admission revealed leukocytosis (white blood cell count, 28,800/*µ*L) with an increase in the level of eosinophils (17.0%) and plasma cells (11.0%); anemia (hemoglobin level, 10.0 g/dL); decreased percentage of reticulocytes (2.0%); elevated serum levels of lactate dehydrogenase (LDH) (991 IU/L), creatinine (1.56 mg/dL), and C-reactive protein (4.75 mg/dL); and polyclonal gammaglobulinemia (immunoglobulin (Ig) *G*, IgA, and IgM levels were 5,366 mg/dL, 378 mg/dL, and, 660 mg/dL, respectively). Serum and urinary protein electrophoresis did not reveal a localized band. A clonally rearranged Ig heavy chain JH gene was not detected. Whole-body CT showed lymphadenopathies in the bilateral cervical, subclavian, axillary, mediastinal, para-abdominal aortic, and bilateral inguinal regions; splenomegaly; and a ground-glass pattern in both lungs. A bone marrow examination was performed and it revealed no evidence of malignant cell invasion. However, flow cytometry analysis revealed an increase in the level of plasma cells (accounting for 25.8% of all nucleated cells) with no restriction of light chains. The dosage of PSL was up to 80 mg on the first hospital day, but the treatment was switched to dexamethasone at a dose of 40 mg daily for 3 days on the second hospital day because PSL failed to improve the lymphadenopathies. Biopsy of the left axillary lymph nodes was performed again on the fourth hospital day. A diagnosis of AITL accompanied by reactive plasmacytosis was made based on the results of the two lymph node biopsies and other aforementioned tests. The Ann Arbor stage was IIIB. The international prognostic index was high-intermediate risk. Treatment with subcutaneous administration of bortezomib at a dose of 1.3 mg/m^2^ was started on the fourth hospital day, but the patient experienced chest discomfort on the fifth hospital day. Electrocardiography showed an elevated ST segment elevation in aVf and V2–V4. The patient's serum creatine phosphokinase-MB level was increased to 115 IU/L. These findings led to a diagnosis of acute myocardial infarction (AMI). The patient was admitted to the intensive care unit. Percutaneous coronary angioplasty was performed, and antiplatelet therapy with aspirin at a dose of 200 mg and prasugrel at a dose of 20 mg daily and anticoagulant therapy with intravenous administration of heparin were started. An intra-aortic balloon pump was introduced. Chemotherapy with bortezomib was temporarily discontinued. Chest radiography showed shadows in both lungs, which were extended on the seventh hospital day. On the 11th hospital day, the patient experienced respiratory failure, resulting in the need for artificial respiration followed by prone positioning. Bronchoscopy was performed, which revealed that there were no mucosal abnormalities, lesions, or obstructions in the airways. Serial bronchoalveolar lavage was performed with the sequential administration of normal saline. The return was erythematous effluent. Based on the bronchoscopy and chest radiography findings, a diagnosis of respiratory failure due to alveolar hemorrhage was made. Subsequently, antiplatelet therapy was discontinued, but heparin administration was continued because the coronary artery thrombus remained. An artificial cardiac pacemaker was inserted because of the onset of atrioventricular block. On the 14th hospital day, the patient's consciousness level dropped to Glasgow coma scale E_4_M_4_M_5_. A fever of 39.2 °C developed and his ECOG performance status increased to 4. His Karnofsky scale score decreased to 10 points. The patient required noradrenaline administration for hypotension and hemodialysis for acute renal failure secondary to hypotension. The patient received steroid pulse therapy with methylprednisolone at a dose of 1,000 mg for 3 days for alveolar hemorrhage, followed by PSL administration. However, the patient's general condition severely deteriorated, and he required chemotherapy because the developing fever was considered tumor fever secondary to AITL recurrence. Therefore, five doses of bortezomib were administered, following which the tumor fever disappeared and his serum LDH level decreased. On the 20th hospital day, the patient no longer required artificial respiration only hemodialysis. Compared with the CT scans of the 13th hospital day, those of the 30th hospital day showed a reduction in lymphadenopathies (<50% reduction), and the size of the splenomegaly remained the same. The patient's general condition gradually improved. On the 135th hospital day, the patient was discharged and followed up as an outpatient thereafter. After discharge from the hospital, approximately 1 month later, positron-emission tomography revealed ^18^F-fluorodeoxyglucose uptake in the lymph nodes as follows: bilateral cervical region (maximum standardized uptake value [SUV max], 7.5), around the stomach on the lesser curvature side (SUV max, 6.02), and para-abdominal aortic region (SUV max, 8.76). Thus, there were lymphadenopathies with ^18^F-fluorodeoxyglucose uptake as revealed via positron-emission tomography, but they had not progressed. Therefore, the patient did not require chemotherapy for approximately 10 months. However, lymphadenopathies progressed thereafter. The patient had received cytotoxic chemotherapy and died of AITL progression approximately 10 months after starting cytotoxic chemotherapy.

## 3. Discussion

Bortezomib is known to be effective for the treatment of B-cell neoplasms. [7, 8] Moreover, several clinical and in vitro studies have demonstrated the efficacy of bortezomib in the treatment of T-cell neoplasms, as described below.

Regarding the safety of using bortezomib for AITL treatment, a previous phase 2 study reported the outcomes of CHOP therapy plus bortezomib use for the treatment of peripheral T-cell lymphoma. [13] The study was conducted in 46 previously untreated patients with peripheral T-cell lymphomas, including 8 patients with AITL. Twenty-nine patients completed the planned treatment schedule (median number of cycles: 6), and the response was assessed in 44 patients because 2 patients dropped out early as they developed grade 4 hepatic dysfunction and pneumonia. Grade 3–4 leucopenia (common terminology criteria for adverse events (CTCAE) ver 3.0) was the most frequent adverse event, whereas neurotoxicity was tolerable (grade 1–2 peripheral neuropathy). The findings pertaining to the present case suggest the safety of using bortezomib for the treatment of T-cell lymphomas, where the only adverse effect was leukopenia grade 1 (CTCAE ver 5.0). The patient developed AMI the day after bortezomib administration, but it is speculated that AMI was caused by hypercoagulation associated with the administration of a high dose of dexamethasone. Furthermore, the most noteworthy aspect was that chemotherapy with bortezomib was well tolerated despite the patient's severely deteriorated condition.

There are two case reports of bortezomib use in patients with AITL (Table 1). [14, 15] In Case 1, similar to that in our case, bortezomib was used in combination with steroids, which resulted in a reduction in lymphadenopathies. The clinical effectiveness of bortezomib was indicated. In Case 2, because bortezomib was used in combination with other anticancer drugs, it is not clear whether bortezomib was effective for AITL. In our case, the patient did not require chemotherapy for 10 months. This clinical course indicates that bortezomib is a possible treatment option for patients with AITL.

## 4. Conclusion

The present case indicates that bortezomib is a possible treatment option for patients with AITL. Furthermore, it may be well tolerated, even if the patient's general condition deteriorates.

## Figures and Tables

**Table 1:** Previous case reports of bortezomib use in angioimmunoblastic T-cell lymphoma.

	Age/Sex	Treatment	Response	Duration of effectiveness	Reference
case1	63/Female	Bortezomib and dexamethasone	Lymphadenopathies reduced in size	4 months	14

case2	76/Female	Bortezomib, mitoxantrone, and dexamethasone	Disappearance of lymphadenopathy	18 months	15
